# A Neural Network Approach to Identify the Peritumoral Invasive Areas in Glioblastoma Patients by Using MR Radiomics

**DOI:** 10.1038/s41598-020-66691-6

**Published:** 2020-06-16

**Authors:** Jiun-Lin Yan, Chao Li, Anouk van der Hoorn, Natalie R. Boonzaier, Tomasz Matys, Stephen J. Price

**Affiliations:** 1 Brain tumour imaging lab, Division of neurosurgery, Department of clinical neuroscience, University of Cambridge, Addenbrooke’s hospital, Box 167, CB2 0QQ Cambridge, United Kingdom; 2 Department of radiology, University of Cambridge, Addenbrooke’s hospital, Box 218, CB2 0QQ Cambridge, United Kingdom; 3 Department of radiology (EB44), University Medical Centre Groningen, University of Groningen, Box 30.001, 9700 RB Groningen, The Netherlands; 4 Department of neurosurgery, Chang Gung Memorial Hospital, 204 Keelung, Taiwan; 5 Department of Chinese Medicine, Chang Gung University College of Medicine, 333 Taoyuan, Taiwan

**Keywords:** Cancer imaging, CNS cancer, CNS cancer

## Abstract

The challenge in the treatment of glioblastoma is the failure to identify the cancer invasive area outside the contrast-enhancing tumour which leads to the high local progression rate. Our study aims to identify its progression from the preoperative MR radiomics. 57 newly diagnosed cerebral glioblastoma patients were included. All patients received 5-aminolevulinic acid (5-ALA) fluorescence guidance surgery and postoperative temozolomide concomitant chemoradiotherapy. Preoperative 3 T MRI data including structure MR, perfusion MR, and DTI were obtained. Voxel-based radiomics features extracted from 37 patients were used in the convolutional neural network to train and as internal validation. Another 20 patients of the cohort were tested blindly as external validation. Our results showed that the peritumoural progression areas had higher signal intensity in FLAIR (p = 0.02), rCBV (p = 0.038), and T1C (p = 0.0004), and lower intensity in ADC (p = 0.029) and DTI-p (p = 0.001) compared to non-progression area. The identification of the peritumoural progression area was done by using a supervised convolutional neural network. There was an overall accuracy of 92.6% in the training set and 78.5% in the validation set. Multimodal MR radiomics can demonstrate distinct characteristics in areas of potential progression on preoperative MRI.

## Introduction

Glioblastoma (GBM) is the most aggressive and the most common primary brain tumour. Despite the extensive standard multimodal treatment regimens including surgical resection followed by concomitant chemo-radiotherapy, the median overall survival is only about 14.9 month^1^. This poor prognosis largely due to the failure of the local treatment which usually aims the contrast-enhancing lesion as the treatment target. Studies had shown that the GBM cancer cell can extent to the non-contrast enhancing area^2,3^. Therefore, the recurrence is inevitable and even in patients with a total resection of the contrast enhancing tumour and subsequent chemoradiotherapy, recurrence happens in all patients and in up to 90% in or directly adjacent to the resection area^4^.

However, the imaging characteristics to this peritumoural non-enhanced invasive area is still not clear. The conventional structure MRI has numerous limitations on the detection of the peritumoural invasive area. Although a high signal in T2 represents vasogenic oedema that associated with the tumour, however, in a biopsy study a normal signal in conventional T2 MRI can have 4% of false negative rate^5^. Therefore, many different MR techniques have been developed to achieve a better demarcation of the GBM. Apparent diffusion coefficient (ADC) is sensitive to the diffusion of water, and can decrease while the extracellular water diffusion is restricted due to the increase of glioma cellularity^6^. Diffusion tensor imaging (DTI) can detect subtle white matter change caused by the tumour infiltration^7^, further decompose DTI in to isotropic p component and anisotropic q component can be used predict the tumour recurrence type by their pattern^8^. In a recent study focused on the peritumoural abnormal DTI-p areas showed that an increase of the relative cerebral blood volume (rCBV), Choline, Choline/ N-acetyl aspartate (NAA), and the glutamate+ glutathione (Glx)^9^. Another study focused on the DTI fractional anisotropy (FA) in the peritumoural non-enhancing area showed that a decrease of FA may indicate tumour infiltration which further lead to local tumour recurrence^10^.

Radiomics is an emerging technique that can provide a more detailed quantification multi-modal MR study. And is defined as the conversion of images to higher dimensional data and the subsequent mining of these data^11^. In the conventional imaging analysis, only limited semantic features can be extracted, however, by using high-throughput computing, radiomics can quantify more detailed agnostic features including first order, second order or higher order. Hu *et al*., in 2015 used multi-parametric MRI and texture analysis to predict tumour density in both enhanced and non-enhanced part based on a biopsy study and showed 85% accuracy in training set and 81.8% accuracy in the validation^12^. Further study conducted by Kim *et al*., showed that the FA and the CBV radiomics of the peritumoural area, together with the clinical index, can potentially predict the tumour progression^13^.

Thus, in this study we aimed to characterize the preoperative peritumoural non-enhanced area that demonstrated to have generated tumour progression later on. And further applied these features, together with radiomics features to identify areas of GBM progression on the preoperative MRI by using the convolutional neural network.

## Materials and Methods

### Patient inclusion criteria

We retrospectively included 57 patients with a newly diagnosed cerebral glioblastoma in this study. Exclusion criteria were previous cranial surgery, previous cerebral radiotherapy or a known other primary tumour. General characteristics were shown in Table 1. 37 patients who had follow-up MRI in Cambridge university hospital were used for training and internal validation. Another 20 patients who received follow-up MRI in other hospital were collected as external validation. There were 5 patients in the training group had radiological pseudo-progression during the follow-up, but all patients had tumour progression later. Tumour location was classified according to a previous classification^14^. All patients had a Karnofsky performance status ≥70 and were treated with a maximal surgical resection using neuronavigation (StealthStation, Medtronic) and 5-aminolevulinic acid (5-ALA) fluorescence guidance with the aim of maximal tumour resection. This was followed by standard concomitant chemoradiotherapy and adjuvant chemotherapy^15^.

**Table 1:** General Characteristics of the Patients.

	Training Group	Validation Group	Pseudo-progression	***p***-value
Total number of patients	37	20	5	
Males/ females	24/ 13	14/ 6	4/ 1	0.7746
Age (years)	55 ± 12	60 ± 9	55 ± 9	0.2860
Tumour location				0.7807
Eloquent	8	4	0	
Near eloquent	14	9	3	
Non eloquent	15	7	2	
Midline shift (mm)	3.8 ± 3.8	3.3 ± 3.1	2.9 ± 3.6	0.8358
Pre-OP tumour size (mL)^a^	44 ± 25	39 ± 26	40 ± 14	0.7573
GTR/ STR^a^	29/ 8	13/ 7	5/ 0	0.2228
PFS (median, days)	262	181	778	0.0104
OS (median, days)	523	407	864	0.0144
MGMT un-methylated	16	4	1	0.0958
methylated	10	9	3	
IDH-1 wild type	34	17	4	> 0.9999
mutated	3	1	1	

^a^The pre-operative tumour volume and the extent of resection were evaluated based on the contrast enhanced T1-weighted MRI.

GTR = gross total resection; STR = subtotal resection; PFS (progression free survival); OS = overall survival; MGMT = O^6^-methylguanine DNA methyltransferase; IDH-1 = Isocitrate dehydrogenase.

The study protocol was approved and regulated by the National Research Ethics Service (NRES) Committee East of England - Cambridge Central (ethics reference no. 10/H0308/23). And informed written consents were obtained from all patients and all methods were performed in accordance with the relevant guidelines and regulations.

### MRI Data acquisition and imaging processing

Detailed MRI data acquisition and imaging processing protocols were as previous described^16,17^. In brief, all preoperative MRI data was obtained from the same 3.0 Tesla Siemens MR Magnetron system (Siemens Healthcare, Munich, Germany) with a standard 12-channel head coil. The imaging protocol included anatomical contrast 3D T1-weighted magnetization-prepared rapid acquisition with gradient echo (MPRAGE), T2-weighted and fluid-attenuated inversion recovery (FLAIR) sequences. The preoperative DTI, dynamic susceptibility weighted contrast enhanced (DSC) MR perfusion and ^1^H MR spectroscopy data were also acquired. DTI data was performed using a single-shot echo-planar sequence scanned in 13 directions.

Follow-up MR protocols included a 2D contrast enhanced T1-weighted, a T2 and a FLAIR sequences. Patients may receive the scanning from different scanner according to the clinical arrangement (a 1.5 T GE Optima, 1.5 or 3.0 T GE Signa or 1.5 T Siemens Avanto).

Imaging processing of the preoperative DTI were done by using tools from the FMRIB Software Library (FSL) version 5.0.0 (http://fsl.fmrib.ox.ac.uk/fsl/fslwiki/). DTI was further decomposed into isotropic (p) component and anisotropic (q) component after eigenvalues were calculated in the DTI data as described previously described^18^. Perfusion MR was processed using NordicICE (NordicNeuroLab AS, Bergen, Norway) and maps of rCBV were created following contrast agent leakage correction. Spectroscopy data was processed using LCModel^19^ and the MRS result in each ROIs were obtained by using a previous described 3D voxel-wise approach^17^.

### Regions of interests

**Figure 1:**
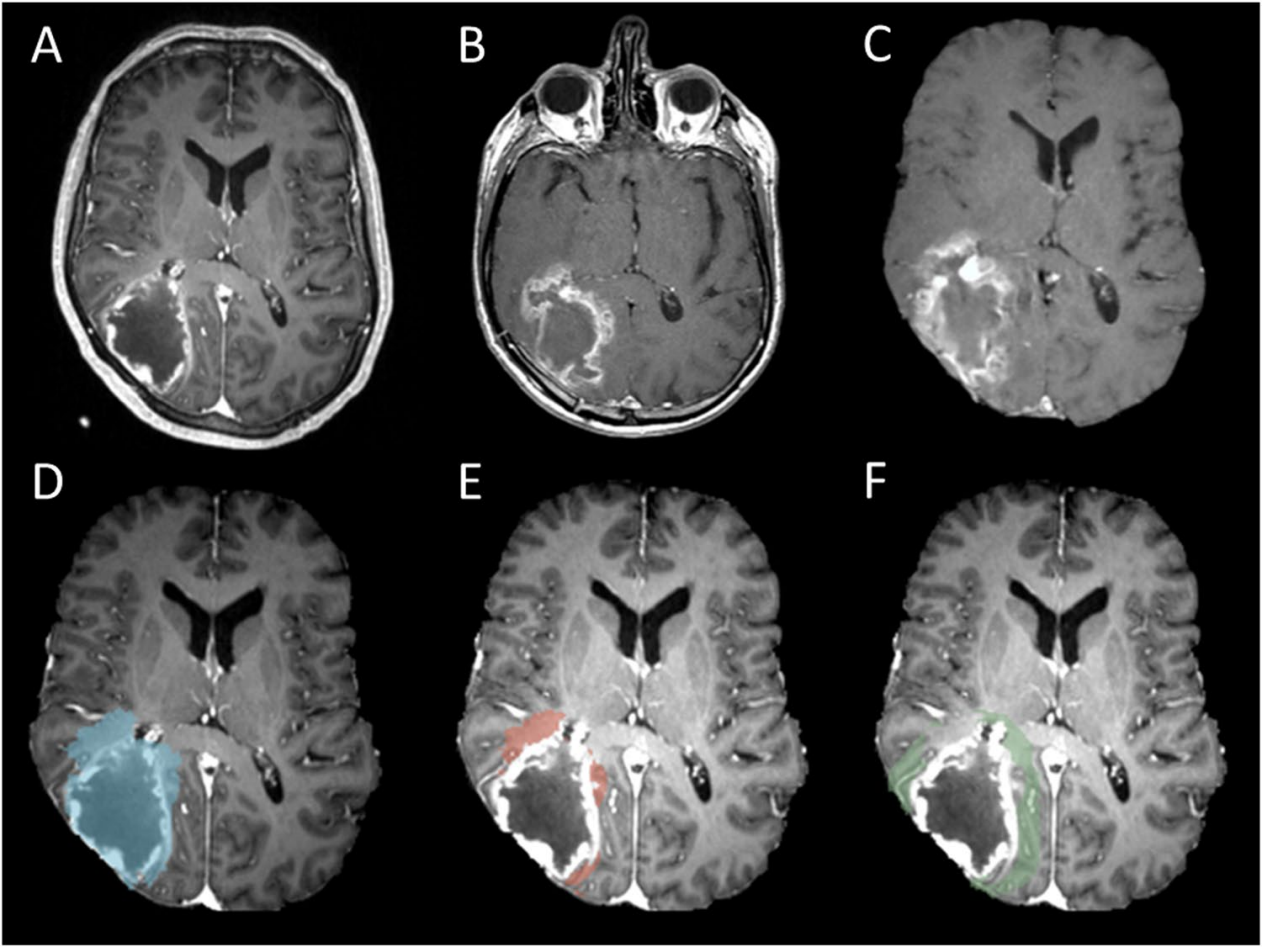
An example of tumour progression (**B**) was coregistered to presurgical image (**A**). The coregistered image (**C**,**D** blue) can further create progression area (**E**, red) and 10 mm non-progression area (**F**, green).

Two main regions of interest (ROIs), progression areas and non-progression areas were created in this study (Fig. 1).

All patients had radiological tumour progression in our cohort. The progression area was drawn from the first tumour progression T1 contrast MRI (Fig. 1B) according to the RANO criteria^20^ by the first authors (J.L.Y.) and approved by the 3^rd^ author (A.H.). In order to avoid pseudoprogression, we examined at least two follow-up scans after the first radiological tumor progression, except for a few patients with rapidly clinical deterioration, and did not receive another follow up scan after the progression.

The areas of the tumour progression (Fig. 1C,D) were created by coregistration the progression contrast enhanced T1 MRI (Fig. 1B) to the pre-operative diagnostic MRI (Fig. 1A). This was done by using a previous described two stage non-linear semi-automatic coregistration^21^. In short, firstly, we calculated the transformation matrix between preoperative tumour and postsurgical resection cavity by using the linear FLIRT co-registration. Then we applied this transformation matrix to a non-linear FNIRT transformation to coregister the brain. After subtraction of the preoperative contrast enhanced lesion, the peritumoural tumour progression (Fig. 1E) and non-progression areas (Fig. 1F) can be identified on the preoperative MRIs.

The non-progression areas were created from the peritumoural 5, 10, 15, 20 mm excluding the progression areas. In addition, a contralateral area of normal appearing with matter (NAWM) as control representing normal brain tissue.

### Radiomics analysis and machine learning

The multimodal MRI characteristics preoperatively are extracted using Matlab (MathWorks Inc., Natick, MA, USA). First order and second order radiomics features were extracted from 7 different MR sequences by using Matlab. The first order features include mean, standard deviation, median, minimum, maximum, variance, skewness, kurtosis, energy, entropy, uniformity, root mean square, mean gray level. The second order features include GLCM (gray level co-occurrence matrix which includes, auto-correlation, contrast, correlation-M, correlation-P, cluster-Prominence, cluster-shad, dissimilarity, energy, entropy, homogeneity-M, homogeneity-P, sum of square, Sum of variance, sum of entropy, difference of Variance, Difference of entropy, Information measure if correlation 1, Information measure of correlation 2, and inversed difference normalised) and GLRLM (gray level run length matrix which includes, short run length, long run length, gray level non-uniformity, run percentage, run length non-uniformity, low gray level emphasis, and high gray level emphasis). A total number of 294 features were extracted.

Identification of the progression areas were done by using supervised convolutional neural network from the Matlab neural network toolbox (nntool). The training of the machine learning was done voxel by voxel. Voxel-wise feature extraction from 37 patients (Table 1, Training group) who had follow-up MRI in Cambridge university hospital were used for training and internal validation. A total number of 2,589,473 peritumoural voxel with 294 radiomics features were input for training. The output was binary, either progression or non-progression. The input data was divided randomly with 70% used for training, 15% for validation, and 15% for testing. There was 10 hidden layers in our neural network, and we used scaled conjugated gradient back propagation in our training. The network performance was calculated by using cross-entropy. The trained model was further tested in another 20 patients who received follow-up MRI in other hospital were collected as external validation (Table 1, validation group). The results were tested 3 repeated time. Further identification of the areas of progression were drawn by using a probability heat map in Matlab.

### Statistical analysis

General patients’ characteristics were tested for group differences with a t-test or Mann-Witney U test for continuous variable depending on the normality of the data. A chi-square test was used for categorical data. Differences between MRI characteristics of the preoperative area later showing tumour progression and non-progression areas were done using paired t-test. Two-sided *p*-values were used. All statistical tests were performed using SPSS version 22 (IBM Inc., New York, USA).

## Results

### Multimodal MRI characteristics

**Figure 2:**
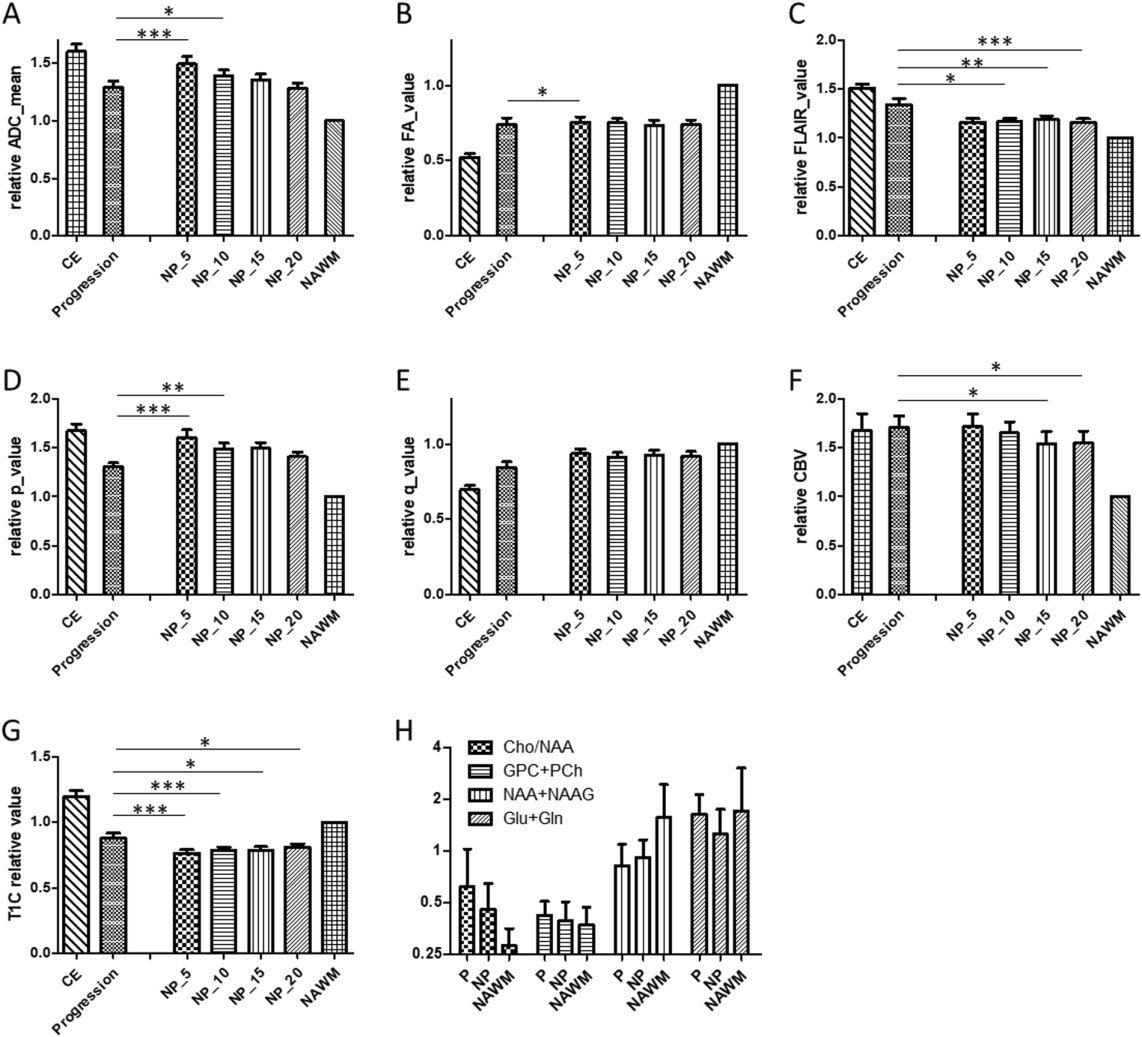
Showed MRI characteristics (**A**–**G**) of contrast enhanced, progression, non-progression (NP) area in 5–20 mm peritumourtumoural area and contralateral normal appearing white matter (NAWM, control). (**H**) Showed MRS of Cho/NAA, Choline, NAA + NAAG and Glu + Gln of progression (P), non-progression (NP) and NAWM. *p < 0.05; **p < 0.01; ***p < 0.001.

Multimodal MRI characteristics were shown in Fig. 2. The ADC values in the progression area were lower than 5 mm and 10 mm peri-tumoural non-progression area (Fig. 2A, p < 0.001, 0.029). Fractional anisotropy (FA) showed lower in progression area than 5 mm non-progression area (Fig. 2B, p = 0.041). The isotropic p component showed a significant decrease in progression area than 5 mm and 10 mm of non-progression area (Fig. 2D, p < 0.001). In areas of progression, anisotropic q did not show difference between progression and non-progression area (Fig. 2E). In areas of later progression, there was significant increase in the FLAIR signal (Fig. 2C, p = 0.020 ~ <0.001) and contrast enhanced T1 MRI (Fig. 2G, p = 0.026 ~ 0.0004) compared to non-progression area. Relative cerebral blood volume was increased in the progression areas than 15–20 mm non-progression area (Fig. 2F, p = 0.038 ~ 0.042). A higher Cho/NAA and lower NAA can be seen in the progression areas but without statistical significance (Fig. 2H)

### Radiomics features

**Figure 3:**
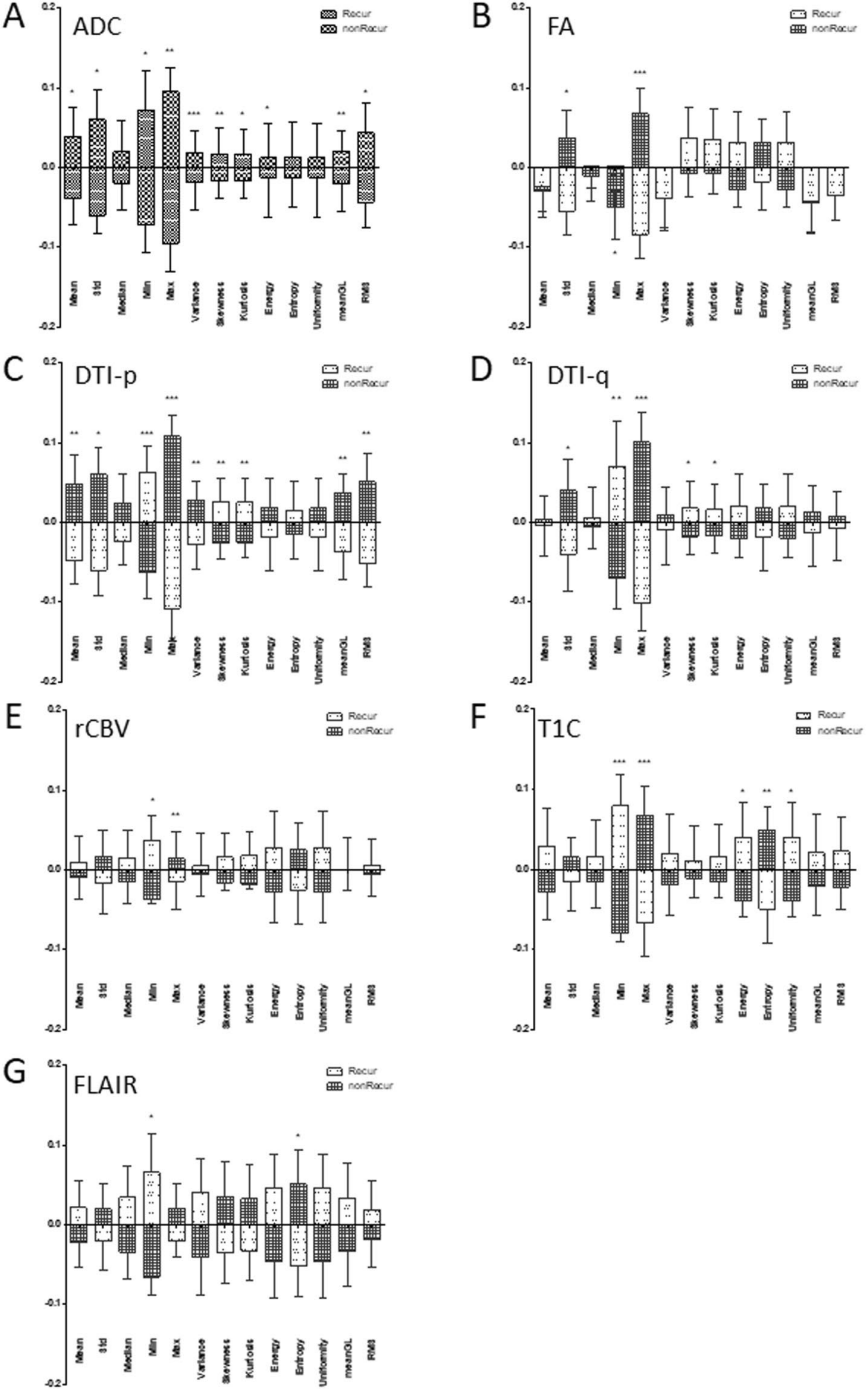
First order radiomics features. Comparison of the first order radiomics features between progression and non-progression area. *p < 0.05; **p < 0.01; ***p < 0.001.

**Figure 4:**
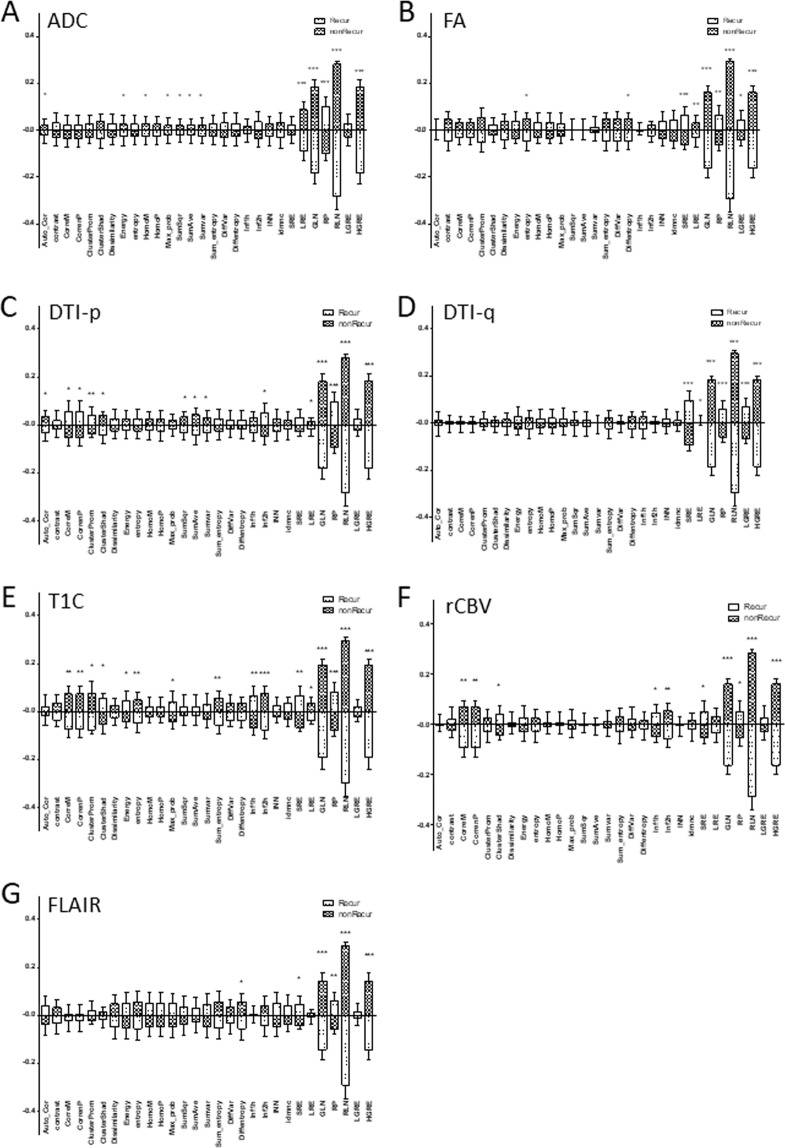
Secondary radiomics features. Comparison of the secondary order radiomics features between progression and non-progression area. *p < 0.05; **p < 0.01; ***p < 0.001.

Thirty-five out of the 91 first order radiomics features had significant difference between progression area and non-progression area (Fig. 3). Most distinct features were in ADC, p and contrast enhanced T1 MRI (Fig. 3A,C,F). In the 203 second order radiomics features, 77 were found to have significant difference between progression and non-progression areas (Fig. 4). Total 112 radiomics features were identified in the progression areas.

### Identification of the progression areas from the pre-operative MRIs

**Figure 5:**
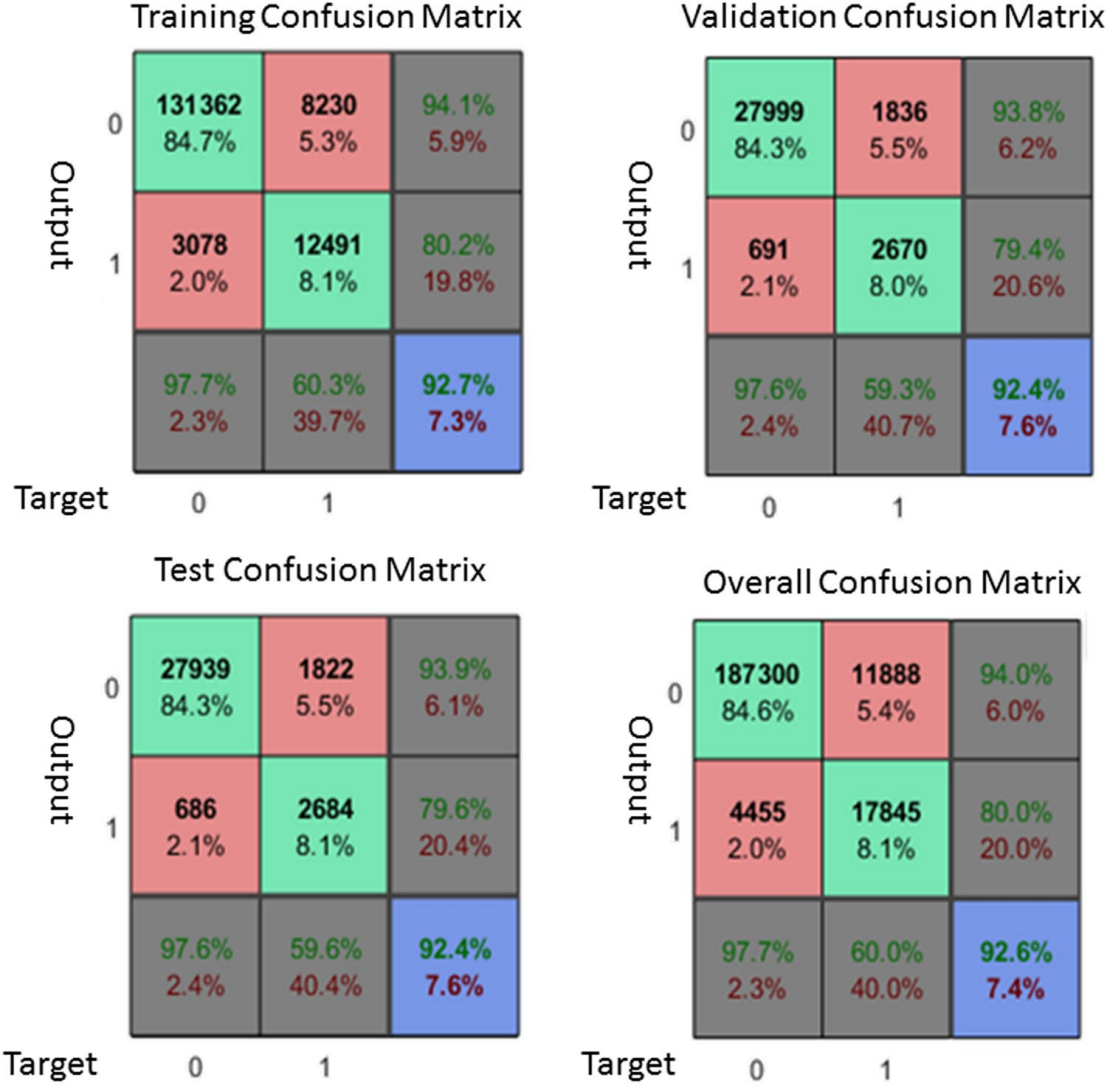
Showed the confusion matrix of the voxel-wise radiomics features in the supervised convolutional neural network model. The overall sensitivity, specificity and accuracy were 80%, 07.7% and 92.6% respectively.

In the training set (n = 37) of the supervised convolutional neural network, the overall accuracy 92.6% (Fig. 5). The training, testing and validation accuracy were 92.7%, 92.4% and 92.4% respectively. The overall sensitivity was 80% and the overall specificity was 97.7%.

**Figure 6:**
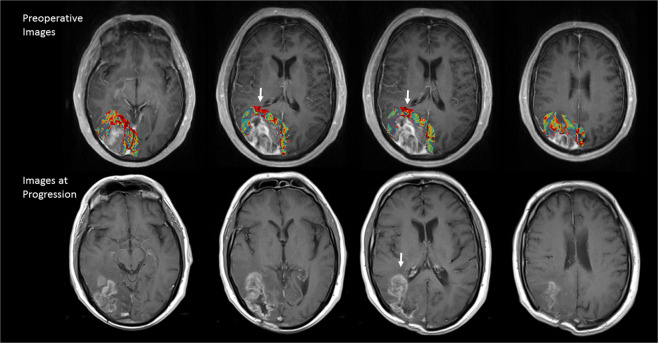
A representative result of the areas of progression (color map) drawn automatically with machine learning and compared to its actual images at progression. The color map showed the probability of progression (red: high, blue: low). White arrows indicate that a high chance of progression at periventricular area.

Further application of the trained model can be used to generate predicted areas of progression on the preoperative MRI, and the example case was shown in Fig. 6.

External validation in the 20 cases were done by overlapping the resulted progression map drawn by the convolutional neural network model and the actual progression MRI. 3 repeated validation results were shown in Table 2. The overall accuracy in the validation group was 78.0%. The positive predict value and the negative predict value were 16.8% and 78.5%.

**Table 2:** External Validation.

	PPV (%)	NPV (%)	Overall Accuracy (%)
Test 1	19.3	78.5	78.0
Test 2	13.4	78.5	78.0
Test 3	17.3	78.5	78.0
	16.8	78.5	78.0

## `Discussion

In this study, we showed distinctive imaging characteristics in the peritumoural areas that potentially progress. Lower ADC, p and higher FLAIR, contrast enhanced T1 signal were noted in these areas. Further comparison by using radiomics features showed more difference supports the identification of these peritumoural invasive areas.

We found a significant lower ADC pixel value in the progression areas this may due to the increase cancer cellularity in this invasive peritumoural areas. ADC is usually used to quantify the diffusivity of the water. In the circumstances of the normal brain tissue or increase extracellular fluid, ADC may increase due to the un-restriction of the water diffusion. On the other hand, ADC may decrease due to increase cellularity which resulted to the restriction of the extracellular fluid diffusion^22^. ADC can be seen in higher grade glioma^6^ resulted in a worse prognosis. In addition, an increase in ADC after treatment can be a favourable prognostic predictor^23^.

DTI p is another representative form of the mean diffusivity. Therefore, similar results with the ADC were expected. Previous biopsy had shown an increase of 10% in the p can show tumour infiltration and further MRS and rCBV study also showed that in these p abnormal peritumoural area can have higher Cho/NAA ratio and higher rCBV which refers to high cellular turnover rate and angiogenesis. This further indicated cancer cell activity. Our results showed that q also features contributes to the identification of the progression area. In the previous biopsy study, q was found largely represents cancer itself and was found larger than the contrast enhancing lesion in approximate 50% of the GBM. Extent of resection showed that a larger resection area of the abnormal q can result a better prognosis. Further study in these peritumoural abnormal q areas showed that there were increase rCBV and increase Choline which also indicates cancer activity. In this study, we found less features by using DTI-q, this may because there was another half of the cases that had q abnormal areas smaller or equal to the contrast enhancing areas that may potentially masked the difference.

FLAIR and contrast enhanced T1C were the two conventional MRI that was found to have distinct features in our study. Although FLAIR represents the vasogenic edema of the brain and is non-specific. Many studies used the FLAIR to define the non-enhancing peritumoural area and showed that abnormal cancer cell. Lemée *et al*., found that about 1/3 of the histology analysis in the FLAIR areas had cancer cell infiltration. However, the FLAIR may be affected by steroid treatment or antiangiogenic therapy such as Avastin. In our study, we found that although the peritumoural area were non-enhanced, there was higher signal in the progression areas. This can be due to the subtle blood brain barrier interference by the cancer infiltration that beyond human visual detection capability.

In addition to the difference of pixel value extracted from difference MRI sequences. We used quantitative radiomics feature to characterise the peritumoural progression area from the preoperative MRIs. We found a total 112 out of 294 features that are different between progression and non-progression areas. Most studies focused on the features of the contrast enhancing lesion. Gevaert et at, in 2014, showed the radiomics features can correlate with manual radiologist’s Visually Accessible Rembrandt Images features, patient clinical survival. Another study used quantified radiomics data from 121 GBM patients to cluster 3 distinct MR phenotypes, the peri-multifocal, spherical and rim-enhanced. They found each cluster had its different pathway which possibly explain the distinct prognosis.

Our results in the radiomics provide reasonable size of the features to the application of the convolutional neural networks. And total 294 features coded in every voxel generated a large number of the sample size that can be used to train the model. Although the optimal features number and the sample size were not tested in this study. Our attempt to use the convolutional neural network to draw the possible progression map from the preoperative MRI had shown moderately fair results. Although the positive predict value and the sensitivity were lower, however, the specificity and negative predict were good.

We have several limitations in this study. We are not able to take the MRI sampling time into this study. GBM progression may change from time to time, our progression area was created by coregistration of the first true progression image to the reference image. This may underestimate the area of progression. In addition, the follow-up MRI protocols were different in two groups. Although the preoperative MR radiomics were obtained by the same MR protocols, different MR protocol at the time of tumour progression may have slightly effects on the spatial co-registration. Another limitation is the limited sample size. As most of the radiomics study require a large sample size in the machine learning in order to reach a stable training model. Our initial training accuracy was 92.7% then drop to 78% in the external validation. This can be over-fitted in the initial training due to inadequate sample size. Or may due to the difference between training and the external validation groups. The overall survival and progression free survival were both shorter in the external validation group, we cannot eliminate the potential bias between this two groups.

## Conclusion

Multimodal quantitative MR imaging analysis, including structure MRI, perfusion MR and diffusion tensor imaging can demonstrate distinct characteristics in areas of potential later progression on preoperative MRI. Moreover, the application of these imaging features, site of tumour progression can be potentially identified via a trained machine learning model which provides a direction for future study and treatment target.
