# Publisher Correction: A Neural Network Approach to Identify the Peritumoral Invasive Areas in Glioblastoma Patients by Using MR Radiomics

**DOI:** 10.1038/s41598-020-70346-x

**Published:** 2020-08-11

**Authors:** Jiun-Lin Yan, Chao Li, Anouk van der Hoorn, Natalie R. Boonzaier, Tomasz Matys, Stephen J. Price

**Affiliations:** 1 Brain tumour imaging lab, Division of neurosurgery, Department of clinical neuroscience, University of Cambridge, Addenbrooke’s hospital, Box 167, CB2 0QQ Cambridge, United Kingdom; 2 Department of radiology, University of Cambridge, Addenbrooke’s hospital, Box 218, CB2 0QQ Cambridge, United Kingdom; 3 Department of radiology (EB44), University Medical Centre Groningen, University of Groningen, Box 30.001, 9700 RB Groningen, The Netherlands; 4 Department of neurosurgery, Chang Gung Memorial Hospital, 204 Keelung, Taiwan; 5 Department of Chinese Medicine, Chang Gung University College of Medicine, 333 Taoyuan, Taiwan

Correction to: *Scientific Reports* https://doi.org/10.1038/s41598-020-66691-6, published online 16 June 2020

In the original version of this Article, the author Anouk van der Hoorn was incorrectly indexed. This error has now been corrected.

